# Real-world cost-effectiveness analysis of thymoglobulin versus no induction therapy in kidney transplant recipients at low risk of graft loss

**DOI:** 10.1590/2175-8239-JBN-2024-0060en

**Published:** 2024-12-20

**Authors:** Adrieli Barros Bessa, Marina Pontello Cristelli, Claudia Rosso Felipe, Renato Demarchi Foresto, Marcelo Cunio Machado Fonseca, Jose Medina Pestana, Helio Tedesco-Silva

**Affiliations:** 1 Universidade Federal de São Paulo, Escola Paulista de Medicina, São Paulo, SP, Brazil.; 2 Novartis, São Paulo, SP, Brazil.; 3 Fundação Oswaldo Ramos, Hospital do Rim, São Paulo, SP, Brazil.; 4 Universidade Federal de São Paulo, Departamento de Ginecologia, Núcleo de Avaliação em Tecnologias em Saúde, São Paulo, SP, Brazil.

**Keywords:** Cost-effectiveness, Real-world, Rabbit Antithymocyte Globulin, Kidney Transplantation

## Abstract

**Background:**

A new induction therapy strategy of a single 3 mg/kg dose of rabbit antithymocyte globulin (r-ATG) showed a lower incidence of acute rejection.

**Methods:**

The objective of this study was to use real-world data to determine the incremental cost-effectiveness ratio (ICER) of r-ATG induction for the prevention of acute rejection (AR) in the first year following kidney transplantation and for kidney graft survival over 1, 4, and 10 years of post-transplantation from the perspective of the national public healthcare system. A Markov state transition model was developed utilizing real-world data extracted from medical invoices from a single center. The study population consisted of adults at low immunological risk undergoing their initial transplantation and received kidneys from either living or deceased donors. The intervention of r-ATG induction was compared to no induction. The clinical outcomes considered for this analysis were acute rejection, cytomegalovirus infection/disease, death, graft loss, and retransplantation.

**Results:**

The cost-effectiveness analysis in the first year revealed that the r-ATG group was more cost-effective, with an ICER of US$ 399.96 per avoided AR episode, an effectiveness gain of 0.01 year in graft survival and a total incremental cost of US$ 147.50. The 4- and 10-year analyses revealed an effectiveness gain of 0.06 and 0.16 years in graft survival in the r-ATG induction group, and a total incremental cost of US$ −321.68 and US$ −2,440.62, respectively.

**Conclusion:**

The single 3 mg/kg dose of r-ATG is cost-effective in preventing acute rejection episodes and dominant in the long term of transplantation, conferring survival gain.

## Introduction

The incidence of acute rejection after kidney transplantation has reduced in recent years, although it is still a major risk factor associated with graft loss. Despite the use of more effective immunosuppressive regimens, the incidence of early acute rejection within the first year after transplantation is primarily influenced by the type of induction agent^1,2^. The anti-thymocyte globulin (r-ATG) induction therapy is associated with lower rates of acute rejection compared to basiliximab or other induction therapies among kidney transplant recipients^3,4,5,6^.

The use of r-ATG also evolved recently, with a progressive reduction of its total dose. According to the last consensus and regulatory approval, the typical dose of r-ATG is 6 mg/kg^7^, although not all patients ultimately receive the full dose regimen. In a recent prospective trial conducted in our center, the use of a single 3 mg/kg dose of r-ATG was associated with a reduced incidence of acute rejection compared to basiliximab induction^8^. This observation changed the standard immunosuppressive protocol beginning on June 17, 2014, when all kidney transplant recipients started receiving the single 3 mg/kg dose of r-ATG. Overall, there were no detectable safety concerns. There was no loss of efficacy (similar incidence of acute rejection and graft loss) in patients at higher risk of graft loss previously receiving 6 mg/kg compared to those receiving 3 mg/kg after changing the standard immunosuppressive protocol. Importantly, in patients at lower risk of graft loss previously receiving no induction therapy, the incidence of first-treated acute rejection decreased from 50.6% to 13.7% among patients receiving 3 mg/kg r-ATG^9^.

In this context, the opportunity arouse to perform a real-world pharmacoeconomic analysis of the new induction therapy strategy compared to no induction in kidney transplant recipients in patients at lower risk of graft loss. Hence, this investigation aimed to establish the incremental cost-effectiveness ratio for preventing acute rejection episodes within the first year post-transplantation and for kidney graft survival at 1, 4, and 10 years following transplantation, viewed from the standpoint of the national public healthcare system.

## Methods

The information in this report adheres to the reporting guidelines outlined in the Consolidated Health Economic Evaluation Reporting Standards 2022 (CHEERS) statement^10^.

### Setting and Location

The Brazilian healthcare system (Sistema Único de Saúde – SUS) ensures healthcare access to all Brazilian citizens. Brazil has one of the world’s largest kidney transplant programs, with 6,283 transplants performed in 2019, with over 90% facilitated through the SUS^11^. The SUS covers organ donation and transplant procedures, follow-up care, and immunosuppressive medications. Our transplant center in São Paulo is the largest kidney transplant center in Brazil, performing over 900 kidney transplants per year^12^. A health economic analysis plan was prepared for the respective study and archived in hospital databases.

### Target Population

This study is grounded in real-world evidence and encompassed adult recipients of kidney transplants, whether from living or standard deceased donors, who received induction therapy with 3 mg/kg r-ATG starting June 17, 2014, within our institution. Consequently, a retrospective cohort was assembled, comprising consecutive adult kidney transplant recipients classified as low immunological risk and maintained on immunosuppressive therapy with tacrolimus (TAC), azathioprine (AZA), and prednisone (PRED). Between June 17, 2014 and September 16, 2015, 1,126 kidney transplants were conducted, with 466 in the induction group (r-ATG 3 mg/kg). For the control group (no induction), we identified 466 consecutive kidney transplants that did not undergo induction therapy between June 15, 2014 and January 03, 2013.

These cohorts were characterized by a negative complement-dependent cytotoxicity crossmatch, a panel-reactive antibody (PRA) level below 50%, absence of preformed A, B, and DR donor-specific antibodies with mean fluorescence intensity exceeding 1500, and receipt of an ABO-compatible renal allograft. We excluded kidney transplant recipients with pretransplant seropositivity for human immunodeficiency virus (HIV), hepatitis C virus (HCV), or hepatitis B virus surface antigen (HBsAg), as well as those who underwent simultaneous pancreas-kidney or pediatric transplantation. Recipients who received induction therapy with basiliximab or initial immunosuppressive regimens comprising cyclosporine, everolimus, or mycophenolate were also excluded.

The groups receiving and not receiving induction therapy with r-ATG 3 mg/kg had similar age, sex ratio, race, panel-reactive antibody (PRA) levels, duration of dialysis, and total number of HLA incompatibilities. Table 1 provides a comprehensive table detailing demographic characteristics.

**Table 1:** Demographic characteristics

	No induction	r-ATG 3 mg/kg	p-Value
	(N = 466)	(N = 466)	
Age (years), median (IQR)	44.0 (33.0, 55.0)	43.0 (31.0, 54.0)	0.230
Male sex, %	61.4	66.1	0.130
Race, %			0.800
*White*	43.3	40.6	
*Black*	15.5	16.1	
*Other*	41.2	43.3	
Cause of end-stage renal disease, %			0.190
*Chronic glomerulonephritis*	24.5	20.0	
*Hypertension*	9.0	9.9	
*Diabetes mellitus*	11.2	14.4	
*Polycystic kidney disease*	6.9	8.6	
*Unknown*	39.1	40.6	
*Other*	9.4	6.7	
Type of renal replacement therapy, %			0.170
*Hemodialysis*	82.8	87.1	
*Peritoneal*	4.3	4.3	
*Preemptive*	9.2	5.6	
*Hemodialysis + peritoneal*	3.6	3.0	
Time on dialysis (months), median (IQR)	27.4 (11.0-49.0)	28.1 (14.2-50.8)	0.370
PRA class I, median (IQR)	0.0 (0.0-0.0)	0.0 (0.0-0.0)	0.600
PRA class II, median (IQR)	0.0 (0.0-0.0)	0.0 (0.0-0.0)	0.300
HLA mismatches, median (IQR)	3 (2-3)	2 (2-3)	0.066
CMV IgG serology, %			0.003
*D+/R+*	86.1	85.6	
*D+/R*−	6.0	10.7	
*D*−*/R+*	6.4	3.2	
*D*−*/R*−	1.5	0.4	
Donor age (years), median (IQR)	44.0 (33.0, 51.0)	42.0 (33.0, 49.0)	0.150
Donor male sex, %	51.1	56.2	0.110
Donor type, %			0.080
*Living*	38.8	33.3	
*Deceased*	61.2	66.7	
*Cause of death, %*			0.570
*Cerebrovascular*	45.3	42.8	
*Trauma*	42.8	46.9	
*Other*	11.9	10.3	
Cold ischemia time (hours), median (IQR)	21 (18-26)	22 (19-28)	<0.001
Health Insurance, %	23.0	21.8	0.753
SUS, %	77.0	78.2	

Abbreviations: N: number; r-ATG: rabbit antithymocyte globulin; IQR: interquartile range; PRA: panel reactive antibody; HLA: human leukocyte antigen; CMV: cytomegalovirus; D: donor; R: recipient; SUS: Sistema Único de Saúde (Brazilian public healthcare system).

### Study Perspective

This study was conducted from the perspective of the Brazilian Unified Health System (SUS), considering the resource utilization guidelines and costs specific to the transplant center. Despite the single-center nature of this study, the SUS guidelines and reimbursement values are established by federal policies, so the results may be extrapolated to other centers performing kidney transplants through the SUS.

### Comparators

We compared patients that received the induction therapy with a single 3 mg/kg r-ATG dose (experimental group) with those who did not receive induction therapy (control group). All patients received the same maintenance immunosuppressive therapy consisting of TAC, AZA, and PRED.

### Time Horizon

We established time horizons of 1, 4, and 10 years following kidney transplantation. We analyzed real-world data encompassing relevant factors influencing long-term follow-ups, such as acute rejection and cytomegalovirus (CMV) infection, during the first year of a kidney transplant. The density incidence of CMV infection in this population after 90 days of kidney transplantation is 5.4 episodes/1000 person-years^13^. In our previous study including 4489 patients, biopsy-confirmed late acute rejection free-survival was 97.4, 96.2, 94.7, 94.3, and 93.7% at the end of each of the first 5 years after transplantation^14^. Considering these data, the occurrence of acute rejection and CMV infection after one year is minimal, with negligible impact on both graft and patient survival.

We evaluated real-world graft and patient survival data and retransplants up to 4 years of follow-up after transplantation. We designed a Markov model for the 4- and 10-year time horizons.

### Discount Rate

For the 1-year time analysis, we did not apply a discount rate. For the 4- and 10-year time horizons, we used a 5% discount rate.

### Outcomes and Measurement of Effectiveness

The incremental cost-effectiveness ratio was determined by assessing the reduction in acute rejection episodes during the first year, when they are more prevalent, and the increase in years of graft survival at 4- and 10-years post-transplantation. Acute rejection and graft survival are critical outcomes in kidney transplantation.

### Resource use and Treatment Costs

The analysis considered health resource costs encompassing direct medical costs, which involve medical resources utilized directly in patient treatment, immunosuppression costs, management of adverse events, and patient follow-up after kidney transplantation. However, indirect or nonmedical direct costs, such as patient transportation, were not included in the evaluation. We calculated immunosuppression costs based on the patients’ mean dosing regimens during the first year of kidney transplantation. All costs were converted from Brazilian reals (R$) to US dollars using the average exchange rate on January 9, 2023 (US$ 1 = R$ 5.12) (Table S1).

We obtained costs for the center from the SUS reimbursement table. Based on the costs of treatment, medical consultation, clinical laboratory tests, biopsy, and hospitalization, we computed the average medical expenses per event within each treatment group. Exclusively for the graft loss event, we considered a fixed cost for complications in chronic kidney disease patients on dialysis as this is the exact value of reimbursement our facility receives for this event, although it is not representative of the budget impact. The clinical outcome of death is an absorbing health state. No costs were incurred for it as we considered that all costs had already been calculated based on the costs of treating the other selected clinical outcomes before death (Table 2).

**Table 2:** Total average costs per group (US$)

	Costs	No induction (N = 466)		r-ATG (N = 466)	
		N	Total costs	N	Total costs
r-ATG	255.85	466	–	466	119,226.10
Immunosuppression	2,627.71	466	1,224,512.86	466	1,224,512.86
Follow-up care	510.60	466	237,939.60	466	237,939.60
CMV	412.70	127	52,412.90	157	64,793.90
Acute Rejection	364.46	236	86,012.56	64	23,325.44
Graft loss	15.77	21	331.17	13	205.01
Total costs			1,601,209.09		1,670,002.61

Abbreviations: N: number of patients; r-ATG: rabbit antithymocyte globulin; CMV: cytomegalovirus.

### Model Choice and Structure

We used the incremental cost-effectiveness ratio (ICER) to compare the two strategies because we expected cost and outcome differences between them. ICER is defined as the difference in cost between the two strategies (cost of induction therapy with r-ATG – the cost of not using induction therapy) over the difference in clinical benefit (outcomes) between the two strategies (clinical benefit of induction with r-ATG - clinical benefit of not using induction) according to the following formula:


ICER= (Cost of induction therapy with r-ATG–Cost of not using induction therapy) 

(Effectiveness of induction therapy with r-ATG–Effectiveness of not using induction therapy)

This model compares the direct medical costs and clinical outcomes of a single r-ATG dose as induction therapy against no induction in adults undergoing kidney transplantation. A Markov model was developed to estimate the costs and outcomes of each treatment from year 4 to year 10 following kidney transplantation^15^.

**Figure 1:**
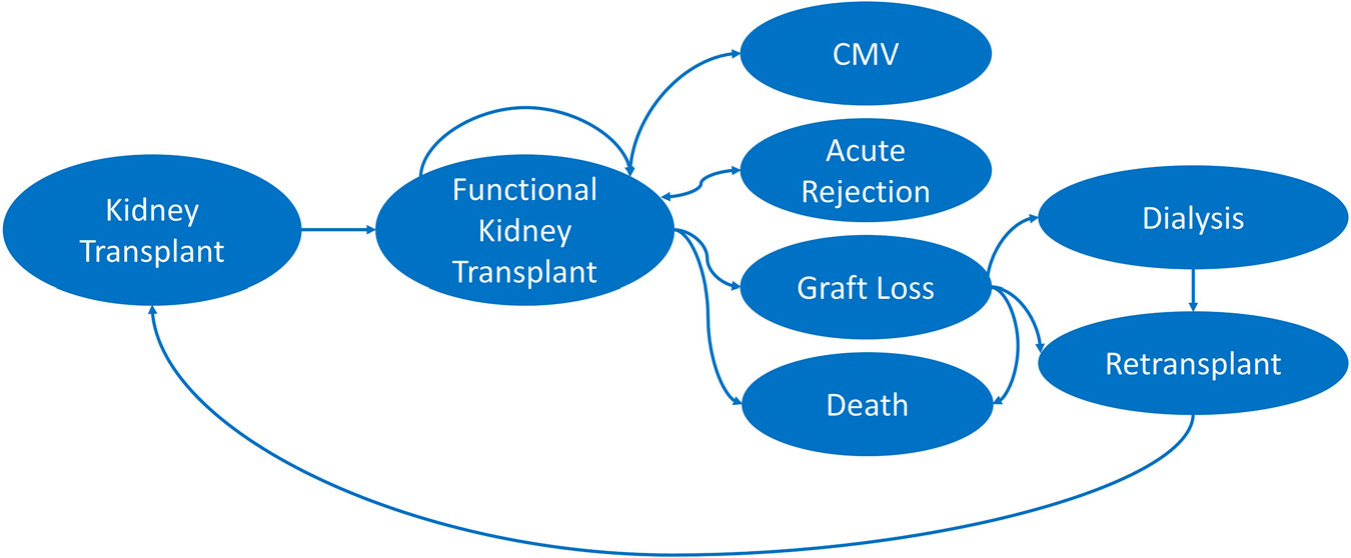
Model structure of health-related events in renal transplant recipients.

The model can track the most clinically and economically relevant events after kidney transplantation, including acute rejection, CMV infection, graft loss, return to dialysis, retransplantation, and death. The model assumes that patients start receiving immunosuppression promptly after transplantation. Patients enter the model in good health with a functioning kidney. Throughout the annual simulation cycles, patients can move to different health states (CMV infection, acute rejection, graft loss, and death). Death is considered a possible outcome in every health state within the model. If the patient does not die, they will recover. Patients who lose their grafts must return to dialysis until they receive a new transplant or die (Figure 1).

The yearly occurrence of clinical outcomes within each treatment group was evaluated based on real-world data up to 4 years post-transplantation (Table 3).

**Table 3:** Incidence of clinical events included in the Markov model

N (%)	Year 1		Year 2		Year 3		Year 4+	
	No induction	r-ATG	No induction	r-ATG	No induction	r-ATG	No induction	r-ATG
CMV infection/disease	127 (27.3)	157 (33.7)	–	–	–	–	–	–
Acute rejection	236 (50.6)	64 (13.7)	–	–	–	–	–	–
Graft loss (with previous AR)	15 (3.2)	6 (1.3)	4 (0.9)	1 (0.2)	4 (0.9)	2 (0.4)	3 (0.6)	3 (0.6)
Graft loss (without previous AR)	6 (1.3)	7 (1.5)	1 (0.2)	2 (0.4)	5 (1.1)	6 (1.3)	2 (0.4)	6 (1.3)
Death with functioning graft	7 (1.5)	13 (2.8)	4 (0.9)	2 (0.4)	2 (0.4)	5 (1.1)	4 (0.9)	3 (0.6)
Death after graft loss	4 (19.1)	0	1 (4.8)	1 (7.7)	1 (4.8)	1 (7.7)	3 (14.3)	2 (15.4)
Retransplantation	0	0	1 (4.8)	0	0	0	2 (9.5)	0

Abbreviations: r-ATG: rabbit antithymocyte globulin; CMV: cytomegalovirus; AR: acute rejection.

After 4 years of kidney transplantation, we considered for the Markov model the incidences according to bibliographical references as described in Table S2. The incidences annualized were calculated through an adjustment by the Poisson model with the formula 1 − (1 − λ)^(1/*t*), where λ is the incidence on reference and t is time^13,16,17^.

For each year of simulation, patients were allocated to health states of the model according to the probabilities presented. For each cycle of the model, this distribution of patients per health state is multiplied by the cost of the state, and over the years of simulation, this cost is accumulated to achieve the total cost.

We considered the same incidences for the two groups because there are no different references in the literature, and the rationale of statistical equivalence can be applied. At the end of the study, the impact on ICER between the groups is determined by the cumulative incidence and cost since the first year, when the outcomes and costs are determining factors for difference in 10 years.

A univariate analysis of all parameters influenced by data variability and uncertainty was performed to estimate the reliability of the economic evaluation results. The univariate analysis evaluates the mathematical model of one parameter changing values within a range of the confidence interval of 95%. The main objective is identifying which parameters are critical for the model. The parameter with the highest variation of the proposed values within a 95% CI indicates the variable that most strongly influences the results of the analysis.

### Sensitivity Analysis

For decision-making, we quantified the uncertainty in the economic model and identified the variables driving such uncertainty; therefore, we performed a probabilistic sensitivity analysis. The model is recalculated using a second-order Monte Carlo simulation in the probabilistic sensibility analysis. The program randomly selects a value for each parameter from a given distribution, a beta distribution for the transition probabilities. All costs considered in the analyses were varied by ±10%, utilizing gamma distributions. While arbitrary, this variation is deemed adequate to encompass the average variation of all cost parameters used in the model. The model was executed ten thousand times, and the results are presented in a cost-effectiveness dispersion plan^18,19^.

## Results

### Cost-Effectiveness Analysis

The cost-effectiveness analysis of avoided acute rejection episodes in the first year after transplantation revealed that the group with r-ATG induction was more effective. It avoided 172 acute rejection episodes, but at an additional cost of US$ 68,793.52 (Table 4).

**Table 4:** Cost-effectiveness analysis of avoided acute rejection episodes in the first year after kidney transplantation

	No induction (N = 466)	r-ATG (N = 466)	Incremental
Total Costs (US$)	1,601,209.09	1,670,002.61	68,793.52
Effectiveness (AR episodes)	236	64	172
ICER (US$ per avoided AR episode)			399.96

Abbreviations: N: number of patients; r-ATG: rabbit antithymocyte globulin; ICER: incremental cost-effectiveness ratio; AR: acute rejection.

The cost-effectiveness analysis of graft survival in the first year following kidney transplantation revealed a minimal effectiveness gain of 0.01 years in graft survival in the r-ATG group and a total incremental cost of US$ 147.50.

The cost-effectiveness analysis of long-term graft survival revealed an effectiveness gain of 0.06 and 0.16 years in graft survival in the r-ATG induction group and a total incremental cost of US$ −321.68 and US$ −2,440.62, remaining dominant in 4 and 10 years (Table 5).

**Table 5:** Cost-effectiveness analysis of graft survival in 1, 4, and 10 years after kidney transplantation per patient

	No induction	r-ATG	Incremental
**Year 1**			
Total Costs (US$)	3,436.11	3,583.61	147.50
Effectiveness (graft survival, years)	0.93	0.94	0.01
ICER (US$ per year of graft survival saved)			14,750.00
**Year 4**			
Total Costs (US$)	12,052.15	11,730.47	−321.68
Effectiveness (graft survival, years)	3.19	3.25	0.06
ICER (US$ per year of graft survival saved)			Dominant
**Year 10**			
Total Costs (US$)	27,105.66	24,665.04	−2,440.62
Effectiveness (graft survival, years)	5.98	6.14	0.16
ICER (US$ per year of graft survival saved)			Dominant

Abbreviations: r-ATG: rabbit antithymocyte globulin; ICER: incremental cost-effectiveness ratio.

One-way sensitivity analysis in year 1 showed that the probability of acute rejection (no induction group), CMV infection/disease (no induction group), and costs of r-ATG were the most critical variables that influence result (Table S3). This analysis also showed that probability of CMV infection/disease (r-ATG group) and probability of acute rejection (no induction group) were the most important variables in year 4 and probability of CMV infection/disease (r-ATG group) was the most important variable in year 10 (Table S3).

### Probabilistic Sensitivity Analysis

**Figure 2:**
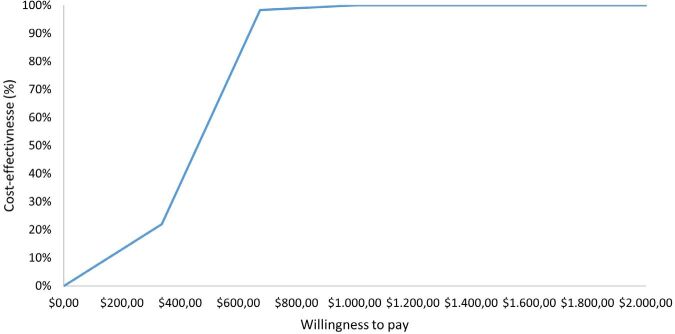
Cost-effectiveness acceptability curve for avoided acute rejection episodes in the first year after kidney transplantation.

Probabilistic sensitivity analysis for avoided acute rejection episodes in the first year after kidney transplantation, represented by the cost-effectiveness acceptability or willingness to pay curves, showed that the cost-effective probability increases significantly from a willingness to pay of around US$ 600 and from a willingness to pay of approximately US$ 1,111; i.e. there is a 100% probability that r-ATG is cost-effective compared to the no induction group (Figure 2).

**Figure 3:**
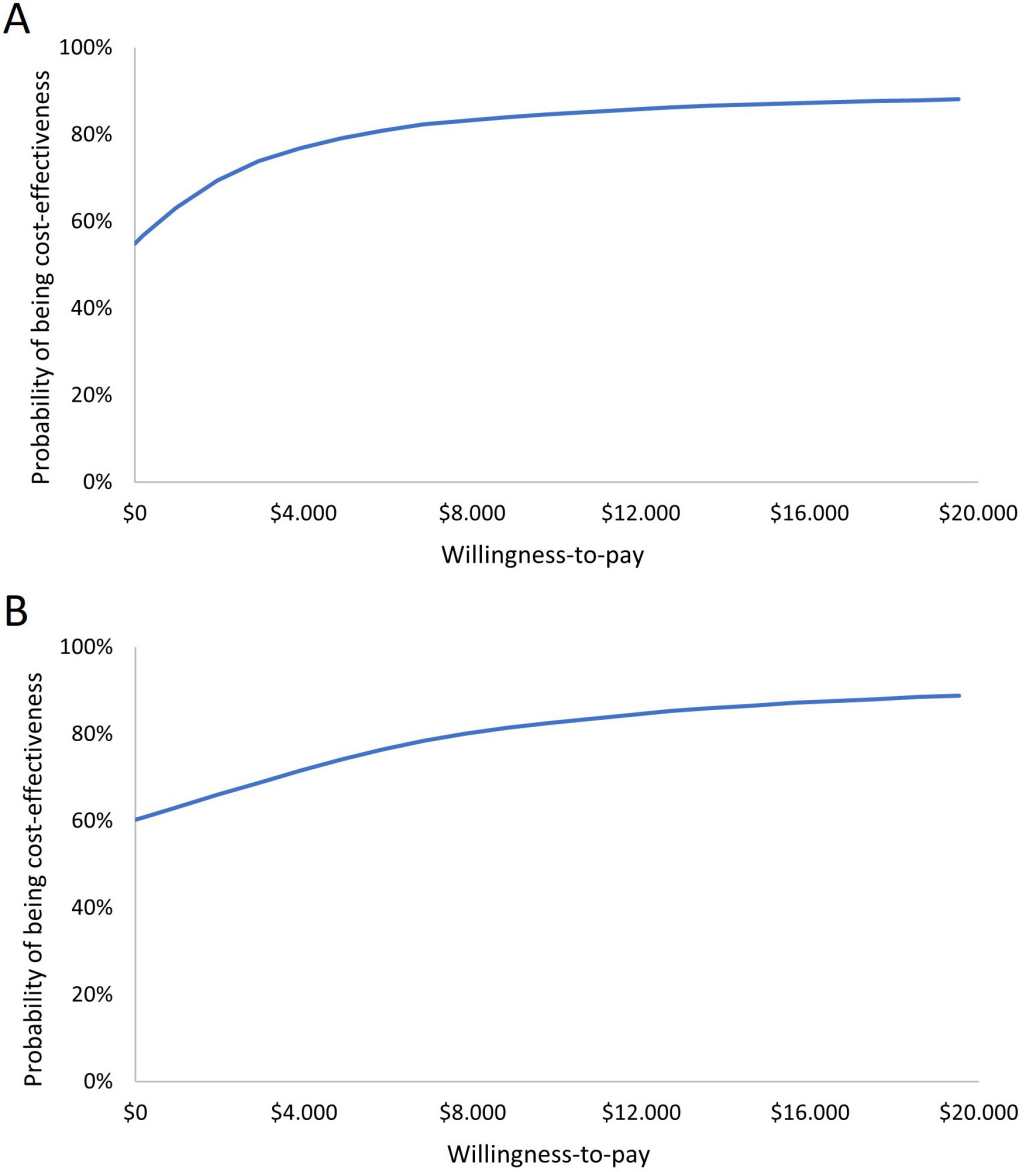
**A.** Cost-effectiveness acceptability curve of graft survival in 4 years after kidney transplantation. **B.** Cost-effectiveness acceptability curve of graft survival in 10 years after kidney transplantation.

Similarly, probabilistic sensitivity analysis for graft survival 4 years after kidney transplantation showed that from a willingness to pay of approximately US$ 6,000, there is more than 80% probability that r-ATG is cost-effective compared to the no induction group (Figure 3A). Finally, corresponding analysis for graft survival in 10 years after kidney transplantation showed that from a willingness to pay of approximately US$ 8,000, there is more than 80% probability that r-ATG is cost-effective compared to the no induction group (Figure 3B).

### Annual Cost Analysis

**Figure 4:**
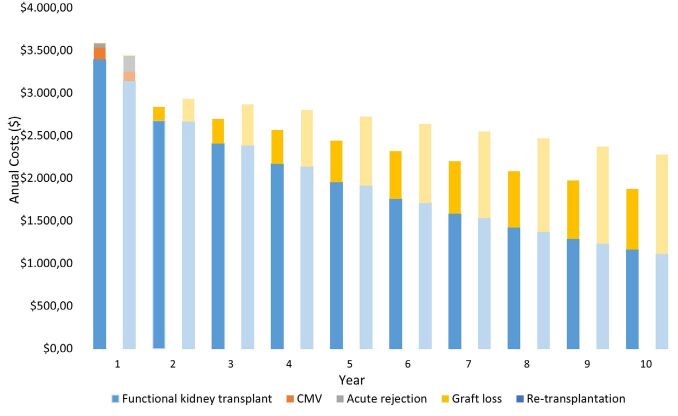
Annual costs for outcomes in r-ATG versus no induction group. Dark color bars represent the r-ATG group and bright color bars represent the no induction group.

Figure 4 describes the annual cost analysis for outcomes in both groups. The first dark-colored bars represent the r-ATG group and the bright-colored bars represent the no-induction group. As described below, the costs for the local health system to maintain functional kidney transplants and complications such as acute rejection and CMV infection were highest in the first year after transplantation. In the following years, the cost profile differed due to graft loss events, such as when the patient returned to dialysis, which featured the primary outcome impacting the health system budget.

## Discussion

The findings of this study hold significant relevance for decision-makers, as the choice of induction therapy can influence both efficacy and cost in the short- and long-term following kidney transplantation. A real-world pharmacoeconomic analysis was performed to discuss a new induction therapy strategy with single 3 mg/kg r-ATG dose compared to no induction in kidney transplant recipients at low risk of graft loss.

Real-world evidence has the potential to drive research on healthcare systems and provide evidence to effectively control future studies, shedding light on how factors like clinical settings, healthcare providers, and health-system attributes influence treatment effects and outcomes. By applying this evidence, we can derive insights pertinent to larger populations, surpassing what might be achievable in specialized research settings. At this moment, there is no real-world pharmacoeconomic analysis of the use of thymoglobulin induction therapy in recipients of kidney transplants in Brazil.

This study aimed to ascertain the incremental cost-effectiveness ratio concerning the prevention of acute rejection episodes within the first-year post-transplantation, as well as the incremental cost-effectiveness ratio related to renal graft survival at 1, 4, and 10 years post-transplantation, from the perspective of the Brazilian public healthcare system.

The r-ATG induction group demonstrated greater effectiveness by averting 172 acute rejection episodes. The incremental cost-effectiveness ratio for this outcome was US$ 14,750 per acute rejection episode avoided. Sensitivity analysis, represented by the cost-effectiveness acceptability curve, showed that with a willingness to pay of approximately US$ 1,111, there was a 100% probability that r-ATG was cost-effective compared to the no induction group. In Brazil, there is no research dedicated to defining a cut-off for willingness to pay. However, the local guide advises that analysis include acceptability curve ranges until the value of one to three times the gross domestic product per capita (GPC) of the country per QALY. In Brazil, the last GPC per capita was US$ 7,946.87, reflecting an acceptable range of willingness-to-pay per episode of acute rejection avoided^20^.

The cost-effectiveness analysis of graft survival revealed an effectiveness gain of 0.01, 0.06, and 0.16 years in graft survival in the r-ATG induction group, respectively at 1, 4, and 10 years after transplantation, being dominant in the 4 and 10-time times. Sensitivity analysis showed a willingness to pay of approximately US$6,000 and US$8,000 for an 80% probability of r-ATG being cost-effective.

One-way sensitivity analysis in year 1 showed that acute rejection and CMV infection/disease are the most critical variables in the model, connected to the risks and benefits of r-ATG therapy.

In Brazil, kidney transplantation is reimbursed through a fixed value for a package of services, including surgery, hospital materials, and medicines; however, the amount does not cover all the costs involved. Two types of induction therapy are listed in this reimbursement: r-ATG (Thymoglobulin^®^) and basiliximab (Simulect^®^), an anti-IL-2 receptor antibody. As per local practice and scientific evidence, r-ATG is used in kidney transplant recipients with low and high risk of graft loss.

Previous analysis investigated other pharmacoeconomic aspects of thymoglobulin induction. A study conducted in the United States evaluated, from the perspective of payers, the incremental cost effectiveness among various agents and approaches to early immunosuppressive treatment in risk-stratified recipients: no-induction, IL2-RA, r-ATG, and alemtuzumab. Gharibi et al.^21^ estimated cumulative costs, graft survival, and the incremental cost-effectiveness ratio (ICER - cost per additional year of graft survival) within three years of transplantation among 19,450 deceased donor kidney transplantation recipients with Medicare as the primary payer from 2000 to 2008^19^. They segmented the study cohort into high-risk individuals (aged > 60 years, panel reactive antibody > 20%, African American ethnicity, Kidney Donor Profile Index > 50%, cold ischemia time > 24 hours), and low-risk individuals (those lacking risk factors, constituting 15% of the cohort). The principal findings revealed no-induction as the least effective and most costly approach. r-ATG and alemtuzumab were deemed more cost-effective across all willingness-to-pay thresholds in the low-risk group, particularly at higher thresholds (US$ 100,000 and US$ 150,000). Notably, the r-ATG group exhibited notably favorable cost-effectiveness acceptability curves (embracing 80% of the recipients) in both risk groups at the US$ 50,000 threshold (excluding those aged > 60 years). Moreover, only r-ATG induction demonstrated a graft survival benefit over the no-induction category (hazard ratio 0.91, 95% confidence interval 0.84 to 0.99) in a multivariable Cox regression analysis^21^.

Morton et al.^22^ evaluated a cost-effectiveness ratio of anti-IL-2 receptor antibody (basiliximab) induction therapy compared to standard therapy without induction or polyclonal antibody (r-ATG). The effectiveness outcome defined in this study was survival, obtaining life-years saved, and quality-adjusted life-years (QALYs). Compared to standard therapy without induction, basiliximab offers a gain of 0.21 life-years saved and 1.42 QALYs, with a cost-saving over 20 years of more than US$ 79,302 per patient treated. The incremental benefits of basiliximab compared to r-ATG induction were 0.35 life-years saved and 0.20 QALYs, with an incremental cost of US$ 5,144 per patient. The incremental cost-effectiveness ratio of basiliximab compared to r-ATG was US$ 14,803 per life-years saved and US$ 25,928 per QALYs^22^.

Another investigation from three German centers evaluated the cost-effectiveness of induction therapies with r-ATG versus basiliximab in kidney transplant recipients. The total average cost of treatment per patient up to the first year after transplantation was € 62,075 and € 59,767 for r-ATG versus basiliximab group (p < 0.01). Therapy with r-ATG exhibited treatment costs similar to basiliximab by the second year, with a predicted cumulative treatment cost savings of € 4,259 under r-ATG compared to basiliximab by the tenth-year post-transplant. The average quality-adjusted life years (QALYs) per patient for one year were 0.809 versus 0.802 for r-ATG and basiliximab, respectively (p = 0.38), with cumulative QALYs of 6.161 and 6.065 per patient by the tenth year^23^.

This study is constrained by its single-center design, specific demographic attributes of the transplant population, and unique characteristics of the Brazilian public healthcare system. Consequently, generalizing findings to populations served by other healthcare systems may be challenging. The model was designed from the SUS perspective and not from the institution perspective. Therefore, costs not covered by the SUS reimbursement packages were not considered. Some important aspects in this population were not considered in the pharmacoeconomic model, such as incidence of BK polyomavirus nephropathy, post-transplant lymphoproliferative disease (PTLD), and hospital readmission for cytopenia. Nonetheless, the reproducibility and analysis of clinical experiences and patient outcomes could be feasible within local healthcare systems and reimbursement frameworks in other countries.

In brief, a single 3 mg/kg r-ATG dose is a cost-effective induction therapy, avoiding acute rejection episodes and conferring survival gain in the long-term after transplantation.

## Supplementary Material

The following online material is available for this article:

Table s1 – Annual costs (US$) of immunosuppression per patient.

Table s2 – Incidence of clinical events included in the Markov model after 4 years.

Table s3 – Univariate analysis.
