# Supplementary material for: Real-world cost-effectiveness analysis of thymoglobulin versus no induction therapy in kidney transplant recipients at low risk of graft loss
Source: J Bras Nefrol. 2024 Dec 20;47(1):e20240060. doi: 10.1590/2175-8239-JBN-2024-0060en (PMC11772011; doi:10.1590/2175-8239-JBN-2024-0060en)
Supplement: Supplementary file 1 [file 2175-8239-jbn-47-1-e20240060-suppl1.pdf]

Braz. J. Nephrol. (J. Bras. Nefrol.)

https://doi.org/10.1590/2175-8239-JBN-2024-0060en

Supplementary Material to “Real-world setting cost-effectiveness analysis of thymoglobulin versus no induction therapy in kidney transplant recipients with low risk for graft loss”

TABLE S1 ANNUAL COSTS (US$) OF IMMUNOSUPPRESSION PER PATIENT.

Immunosuppressive drugs	Mean Dose	Annual Costs	No induction	r-ATG
r-ATG	225 mg	255.85	-	255.85
Tacrolimus	7.4 mg/day	2,559.27	2,559.27	2,559.27
Prednisone	8.6 mg/day	9.98	9.98	9.98
Azathioprine	68.3 mg/day	58.46	58.46	58.46
Total Cost			2,627.71	2,883.56

r-ATG: rabbit antithymocyte globulin.
