# Supplementary material for: Real-world cost-effectiveness analysis of thymoglobulin versus no induction therapy in kidney transplant recipients at low risk of graft loss
Source: J Bras Nefrol. 2024 Dec 20;47(1):e20240060. doi: 10.1590/2175-8239-JBN-2024-0060en (PMC11772011; doi:10.1590/2175-8239-JBN-2024-0060en)
Supplement: Supplementary file 2 [file 2175-8239-jbn-47-1-e20240060-suppl2.pdf]

Braz. J. Nephrol. (J. Bras. Nefrol.)
https://doi.org/10.1590/2175-8239-JBN-2024-0060en

Supplementary Material to "Real-world setting cost-effectiveness analysis of thymoglobulin versus no induction therapy in kidney transplant recipients with low risk for graft loss"

TABLE S2 INCIDENCE OF CLINICAL EVENTS INCLUDED IN THE MARKOV MODEL AFTER 4 YEARS.

Outcome	No induction/ r-ATG	References
Graft loss (without previous AR)	1-(1-5.5%) ^ (1/5) = 1.1%	Jalalzadeh, 2015[13]
Graft loss (with previous AR)	1-(1-63.2%) ^ (1/5) = 18.1%	Jalalzadeh, 2015[13]
Death with a functioning graft	1-(1-10.1%) ^ (1/5) = 2.1%	Cristelli, 2013[14]
Death after graft loss	1-(1-33%) ^ (1/3) = 12.5%	Gill, 2002[15]

r-ATG: rabbit antithymocyte globulin; AR: acute rejection.
