# Supplementary material for: Real-world cost-effectiveness analysis of thymoglobulin versus no induction therapy in kidney transplant recipients at low risk of graft loss
Source: J Bras Nefrol. 2024 Dec 20;47(1):e20240060. doi: 10.1590/2175-8239-JBN-2024-0060en (PMC11772011; doi:10.1590/2175-8239-JBN-2024-0060en)
Supplement: Supplementary file 3 [file 2175-8239-jbn-47-1-e20240060-suppl3.pdf]

Braz. J. Nephrol. (J. Bras. Nefrol.)
https://doi.org/10.1590/2175-8239-JBN-2024-0060en

Supplementary Material to "Real-world setting cost-effectiveness analysis of thymoglobulin versus no induction therapy in kidney transplant recipients with low risk for graft loss"

TABLE S3 UNIVARIATE ANALYSIS.

	ICER VARIATION (US$)		
	**Year 1**	**Year 4**	**Year 10**
Costs of r-ATG (Year 1)	72,089.11	24,958.00	4,233.14
Costs of r-ATG (Year 2)	0	55,239.17	20,849.90
Costs of Immunosuppression (No induction group)	65,692.87	77,785.43	24,357.72
Costs of Acute Rejection	3,353.08	1,100.93	193,69
Costs of Graft loss	2,76	9,39	2,49
Costs of kidney transplantation	0	0	0
Costs of Dialysis	0	4,764.44	3,625.38
Costs of CMV	577,77	260,75	55,74
Costs of Follow-up	0	38,34	67,96
Probability of retransplantation	0	0	0
Probability of CMV infection/disease (no induction group)	172,932.81	12,071.87	4,076.41
Probability of CMV infection/disease (r-ATG group)	38,402.32	241,523.24	1,580,587.33
Probability of Acute rejection (no induction group)	244,904.92	187,865.20	5,354.82
Probability of Acute rejection (r-ATG group)	12,206.24	32,692.66	3,326.19
Probability of Graft loss (no induction group)	17,915.06	66,096.66	4,072.93
Probability of Graft loss (r-ATG group)	5,063.69	2,707.43	3,759.63
Probability of Graft loss with previous AR (no induction group)	0	28,028.47	5,839.23
Probability of Graft loss with previous AR (r-ATG group)	0	14,798,79	1,630.40

Probability of Death (no induction group)	4,703.17	30,956.84	16,167.32
Probability of Death (r-ATG group)	4,703.17	30,227.19	32,306.45
Probability of Death after graft loss (no induction group)	0	0	475.47
Probability of Death after graft loss (r-ATG group)	0	0	0

r-ATG: rabbit antithymocyte globulin; CMV: cytomegalovirus; AR: acute rejection; ICER: incremental cost-effectiveness ratio.
