# Supplementary material for: Real-world cost-effectiveness analysis of thymoglobulin versus no induction therapy in kidney transplant recipients at low risk of graft loss
Source: J Bras Nefrol. 2024 Dec 20;47(1):e20240060. doi: 10.1590/2175-8239-JBN-2024-0060en (PMC11772011; doi:10.1590/2175-8239-JBN-2024-0060en)
Supplement: Translation

# Translation: Análise de custo-efetividade do mundo real da timoglobulina versus ausência de terapia de indução em receptores de transplante renal com baixo risco de perda do enxerto

**DOI:** 10.1590/2175-8239-JBN-2024-0060pt

**Authors:** Adrieli Barros Bessa, Marina Pontello Cristelli, Claudia Rosso Felipe, Renato Demarchi Foresto, Marcelo Cunio Machado Fonseca, Jose Medina Pestana, Helio Tedesco-Silva

**Affiliations:** 1 Universidade Federal de São Paulo, Escola Paulista de Medicina, São Paulo, SP, Brasil.; 2 Novartis, São Paulo, SP, Brasil.; 3 Fundação Oswaldo Ramos, Hospital do Rim, São Paulo, SP, Brasil.; 4 Universidade Federal de São Paulo, Departamento de Ginecologia, Núcleo de Avaliação em Tecnologias em Saúde, São Paulo, SP, Brasil.

**Keywords:** Custo-efetividade, Mundo Real, Globulina Antitimócitos de Coelho, Transplante Renal

## Abstract

**Introdução:**

Nova estratégia de terapia de indução com dose única de 3 mg/kg de globulina antitimócitos de coelho (r-ATG) mostrou menor incidência de rejeição aguda.

**Métodos:**

O objetivo foi utilizar dados do mundo real para determinar a razão de custo-efetividade incremental (RCEI) da indução com r-ATG para prevenção da rejeição aguda (RA) no primeiro ano pós-transplante renal e para sobrevida do enxerto renal em 1, 4 e 10 anos pós-transplante, sob a perspectiva do sistema nacional de saúde pública. Desenvolveu-se um modelo de transição de estado de Markov utilizando dados do mundo real extraídos de faturas médicas de um único centro. A população do estudo consistiu em adultos com baixo risco imunológico submetidos ao primeiro transplante, recebendo rins de doadores vivos ou falecidos. A intervenção de indução com r-ATG foi comparada à não-indução. Os desfechos clínicos considerados para essa análise foram rejeição aguda, infecção/doença por citomegalovírus, óbito, perda do enxerto e retransplante.

**Resultados:**

Análise de custo-efetividade no primeiro ano revelou que o grupo r-ATG foi mais custo-efetivo, com RCEI de US$ 399,96 por episódio de RA evitado, ganho de efetividade de 0,01 ano na sobrevida do enxerto e custo incremental total de US$ 147,50. As análises de 4 e 10 anos revelaram ganho de efetividade de 0,06 e 0,16 anos na sobrevida do enxerto no grupo de indução com r-ATG e custo incremental total de US$ −321,68 e US$ −2.440,62, respectivamente.

**Conclusão:**

A dose única de 3 mg/kg de r-ATG é custo-efetiva na prevenção de episódios de RA e dominante no longo prazo do transplante, conferindo ganho de sobrevida.

## Introdução

A incidência de rejeição aguda após o transplante renal diminuiu ao longo dos últimos anos, embora ainda seja um fator de risco importante associado à perda do enxerto. Apesar do uso de regimes imunossupressores mais eficazes, a incidência de rejeição aguda precoce no primeiro ano após o transplante é influenciada principalmente pelo tipo de agente de indução^1,2^. A terapia de indução com globulina antitimócitos (r-ATG) está associada a taxas mais baixas de rejeição aguda em comparação com basiliximabe ou outras terapias de indução entre os receptores de transplante renal^3,4,5,6^.

O uso de r-ATG também evoluiu recentemente, com uma redução progressiva de sua dose total. De acordo com o último consenso e aprovação regulatória, a dose típica de r-ATG é de 6 mg/kg^7^, embora nem todos os pacientes recebam, em última análise, o regime de dose completo. Em um recente estudo prospectivo realizado em nosso centro, o uso de uma dose única de 3 mg/kg de r-ATG foi associado a uma incidência reduzida de rejeição aguda em comparação à indução com basiliximabe^8^. Essa observação mudou o protocolo imunossupressor padrão a partir de 17 de junho de 2014, quando todos os receptores de transplante renal passaram a receber a dose única de 3 mg/kg de r-ATG. No geral, não foram detectadas preocupações de segurança. Não houve perda de efetividade (incidência semelhante de rejeição aguda e perda do enxerto) em pacientes com maior risco de perda do enxerto, que anteriormente recebiam 6 mg/kg, em comparação àqueles que receberam 3 mg/kg após a alteração do protocolo imunossupressor padrão. É importante ressaltar que, em pacientes com menor risco de perda do enxerto que anteriormente não recebiam nenhuma terapia de indução, a incidência de rejeição aguda no primeiro episódio tratado diminuiu de 50,6% para 13,7% a partir da administração de 3 mg/kg de r-ATG^9^.

Nesse contexto, surgiu a oportunidade de realizar uma análise farmacoeconômica do mundo real, comparando a nova estratégia de terapia de indução com a ausência de indução em receptores de transplante renal nos pacientes com menor risco de perda do enxerto. Dessa forma, esta pesquisa teve como objetivo estabelecer a razão de custo-efetividade incremental para a prevenção de episódios de rejeição aguda no primeiro ano pós-transplante e para a sobrevida do enxerto renal em 1, 4 e 10 anos após o transplante, sob o ponto de vista do sistema nacional de saúde pública.

## Métodos

As informações contidas neste relato seguem as diretrizes de relato estabelecidas na declaração *Consolidated Health Economic Evaluation Reporting Standards 2022* (CHEERS)^10^.

### Cenário e Localização

O sistema de saúde brasileiro (Sistema Único de Saúde - SUS) garante o acesso à saúde a todos os cidadãos brasileiros. O Brasil possui um dos maiores programas de transplante renal do mundo, com 6.283 transplantes realizados em 2019, sendo mais de 90% facilitados por meio do SUS^11^. O SUS cobre os procedimentos de doação e transplante de órgãos, cuidados de acompanhamento e medicamentos imunossupressores. Nosso centro de transplante em São Paulo é o maior centro de transplante renal do Brasil, realizando mais de 900 procedimentos por ano^12^. Um plano de análise econômica da saúde foi elaborado para o respectivo estudo e arquivado nos bancos de dados do hospital.

### População-Alvo

Este estudo se baseia em evidências do mundo real e abrangeu receptores adultos de transplantes renais, provenientes de doadores vivos ou falecidos padrão, que receberam terapia de indução com 3 mg/kg de r-ATG a partir de 17 de junho de 2014, em nossa instituição. Consequentemente, foi montada uma coorte retrospectiva, composta por receptores consecutivos de transplante renal adultos classificados como de baixo risco imunológico e mantidos em terapia imunossupressora com tacrolimus (TAC), azatioprina (AZA) e prednisona (PRED). Entre 17 de junho de 2014 e 16 de setembro de 2015, foram realizados 1.126 transplantes renais, com 466 no grupo de indução (r-ATG 3 mg/kg). Para o grupo controle (sem indução), identificamos 466 transplantes renais consecutivos que não foram submetidos à terapia de indução entre 15 de junho de 2014 e 3 de janeiro de 2013.

Essas coortes foram caracterizadas por uma prova cruzada por citotoxicidade dependente de complemento negativa, nível de painel de reatividade de anticorpos (PRA) inferior a 50%, ausência de anticorpos doador específico A, B e DR pré-formados com intensidade de fluorescência média superior a 1.500, além do recebimento de um aloenxerto renal compatível com o sistema ABO. Foram excluídos os receptores de transplante renal com soropositividade pré-transplante para o vírus da imunodeficiência humana (HIV), vírus da hepatite C (HCV) ou antígeno de superfície do vírus da hepatite B (HBsAg), bem como aqueles submetidos a transplante simultâneo de pâncreas-rim ou transplante pediátrico. Os receptores que receberam terapia de indução com basiliximabe ou regimes imunossupressores iniciais que incluíam ciclosporina, everolimus ou micofenolato também foram excluídos.

Os grupos que receberam e os que não receberam terapia de indução com r-ATG 3 mg/kg apresentaram idade, proporção de sexo, raça, níveis de painel de reatividade de anticorpos (PRA), duração da diálise e número total de incompatibilidades HLA semelhantes. A Tabela 1 apresenta um quadro detalhado das características demográficas.

**Tabela 1:** Características demográficas

	Sem indução	r-ATG 3 mg/kg	Valor de p
	(N = 466)	(N = 466)	
Idade (anos), mediana (IIQ)	44,0 (33,0; 55,0)	43,0 (31,0; 54,0)	0,230
Sexo masculino, %	61,4	66,1	0,130
Raça, %			0,800
*Branca*	43,3	40,6	
*Preta*	15,5	16,1	
*Outra*	41,2	43,3	
Causa da doença renal em estágio terminal, %			0,190
*Glomerulonefrite crônica*	24,5	20,0	
*Hipertensão*	9,0	9,9	
*Diabetes mellitus*	11,2	14,4	
*Doença renal policística*	6,9	8,6	
*Desconhecida*	39,1	40,6	
*Outra*	9,4	6,7	
Tipo de terapia renal substitutiva, %			0,170
*Hemodiálise*	82,8	87,1	
*Peritoneal*	4,3	4,3	
*Preemptiva*	9,2	5,6	
*Hemodiálise + peritoneal*	3,6	3,0	
Tempo em diálise (meses), mediana (IIQ)	27,4 (11,0-49,0)	28,1 (14,2-50,8)	0,370
PRA classe I, mediana (IIQ)	0,0 (0,0-0,0)	0,0 (0,0-0,0)	0,600
PRA classe II, mediana (IIQ)	0,0 (0,0-0,0)	0,0 (0,0-0,0)	0,300
Incompatibilidades HLA, mediana (IIQ)	3 (2-3)	2 (2-3)	0,066
Sorologia CMV IgG, %			0,003
*D+/R+*	86,1	85,6	
*D+/R*−	6,0	10,7	
*D*−*/R+*	6,4	3,2	
*D*−*/R*−	1,5	0,4	
Idade do doador (anos), mediana (IIQ)	44,0 (33,0; 51,0)	42,0 (33,0; 49,0)	0,150
Doador sexo masculino, %	51,1	56,2	0,110
Tipo de doador, %			0,080
*Vivo*	38,8	33,3	
*Falecido*	61,2	66,7	
Causa do óbito, %			0,570
*Cerebrovascular*	45,3	42,8	
*Trauma*	42,8	46,9	
*Outra*	11,9	10,3	
Tempo de isquemia fria (horas), mediana (IIQ)	21 (18-26)	22 (19-28)	<0,001
Seguro de Saúde, %	23,0	21,8	0,753
SUS, %	77,0	78,2	

Abreviaturas: N: número; r-ATG: globulina antitimócitos de coelho; IIQ: intervalo interquartil; PRA: painel de reatividade de anticorpos; HLA: antígeno leucocitário humano; CMV: citomegalovírus; D: doador; R: receptor; SUS: Sistema Único de Saúde.

### Perspectiva do Estudo

Este estudo foi realizado sob a perspectiva do Sistema Único de Saúde (SUS), considerando as diretrizes de utilização de recursos e os custos específicos do centro de transplante. Apesar da natureza unicêntrica deste estudo, as diretrizes do SUS e os valores de reembolso são estabelecidos por políticas federais, de modo que os resultados podem ser extrapolados para outros centros que realizam transplantes renais por meio do SUS.

### Comparadores

Comparamos os pacientes que receberam a terapia de indução com uma única dose de 3mg/kg de r-ATG (grupo experimental) com aqueles que não receberam terapia de indução (grupo controle). Todos os pacientes receberam a mesma terapia imunossupressora de manutenção composta por TAC, AZA e PRED.

### Horizonte de Tempo

Estabelecemos horizontes de tempo de 1, 4 e 10 anos após o transplante renal. Analisamos dados do mundo real abrangendo fatores relevantes que influenciam os acompanhamentos de longo prazo, como rejeição aguda e infecção por citomegalovírus (CMV), durante o primeiro ano de um transplante renal. A densidade de incidência de infecção por CMV nessa população após 90 dias do transplante foi de 5,4 episódios/1000 pessoas-ano^13^. Em nosso estudo anterior, que incluiu 4.489 pacientes, a sobrevida livre de rejeição aguda tardia confirmada por biópsia foi de 97,4, 96,2, 94,7, 94,3 e 93,7% ao final de cada um dos primeiros 5 anos após o transplante^14^. Considerando esses dados, a ocorrência de rejeição aguda e infecção por CMV após um ano é mínima, com impacto insignificante na sobrevida do enxerto e do paciente.

Avaliamos dados do mundo real de sobrevida do enxerto e do paciente, bem como retransplantes de até 4 anos de acompanhamento após o procedimento. Desenvolvemos um modelo de Markov para os horizontes de tempo de 4 e 10 anos.

### Taxa de Desconto

Para a análise temporal de 1 ano, não aplicamos uma taxa de desconto. Para os horizontes de 4 e 10 anos, utilizamos uma taxa de desconto de 5%.

### Desfechos e Medição da Efetividade

A razão de custo-efetividade incremental foi determinada pela avaliação da redução dos episódios de rejeição aguda durante o primeiro ano, quando são mais prevalentes, e do aumento nos anos de sobrevida do enxerto em 4 e 10 anos após o transplante. A rejeição aguda e a sobrevida do enxerto são desfechos críticos no transplante renal.

### USO de Recursos e Custos de Tratamento

A análise considerou os custos de recursos de saúde abrangendo custos médicos diretos, que envolvem recursos médicos utilizados diretamente no tratamento do paciente, custos de imunossupressão, manejo de eventos adversos e acompanhamento do paciente após o transplante renal. No entanto, não foram incluídos na avaliação custos indiretos ou custos não médicos diretos, como o transporte do paciente. Calculamos os custos de imunossupressão com base nos regimes posológicos médios dos pacientes durante o primeiro ano de transplante renal. Todos os custos foram convertidos de reais (R$) para dólares americanos utilizando a taxa de câmbio média de 9 de janeiro de 2023 (US$ 1 = R$ 5,12) (Tabela S1).

Obtivemos os custos para o centro a partir da tabela de reembolso do SUS. Com base nos custos de tratamento, consulta médica, exames laboratoriais clínicos, biópsia e hospitalização, calculamos as despesas médicas médias por evento em cada grupo de tratamento. Exclusivamente para o evento de perda do enxerto, consideramos um custo fixo para complicações em pacientes com doença renal crônica em diálise, uma vez que esse é o valor exato de reembolso que nosso centro recebe por esse evento, embora não seja representativo do impacto orçamentário. O desfecho clínico de óbito é um estado de saúde absorvente. Nenhum custo foi incorrido para ele, pois consideramos que todos os custos já haviam sido calculados com base nos custos de tratamento dos demais desfechos clínicos selecionados antes do óbito (Tabela 2).

**Tabela 2:** Custos médios totais por grupo (US$)

	Custos	Sem indução (N = 466)		r-ATG (N = 466)	
		N	Custos totais	N	Custos totais
r-ATG	255,85	466	–	466	119.226,10
Imunossupressão	2.627,71	466	1.224.512,86	466	1.224.512,86
Cuidados de acompanhamento	510,60	466	237.939,60	466	237.939,60
CMV	412,70	127	52.412,90	157	64.793,90
Rejeição Aguda	364,46	236	86.012,56	64	23.325,44
Perda do enxerto	15,77	21	331,17	13	205,01
Custos totais			1.601.209,09		1.670.002,61

Abreviaturas: N: número de pacientes; r-ATG: globulina antitimócitos de coelho; CMV: citomegalovírus.

### Escolha e Estrutura do Modelo

Utilizamos a razão de custo-efetividade incremental (RCEI) para comparar as duas estratégias, pois esperávamos diferenças de custos e de desfechos entre elas. A RCEI é definida como a diferença de custo entre as duas estratégias (custo da terapia de indução com r-ATG – custo da não utilização da terapia de indução) em relação à diferença no benefício clínico (desfechos) entre as duas estratégias (benefício clínico da indução com r-ATG – benefício clínico da não utilização da indução), de acordo com a seguinte fórmula:


RCEI= (Custo da terapia de indução com r-ATG–Custo da não utilização da terapia de
indução) 

(Efetividade da terapia de indução com
r-ATG−Efetividade da não utilização da terapia de
indução)

Este modelo compara os custos médicos diretos e os desfechos clínicos de uma única dose de r-ATG como terapia de indução versus a ausência de indução em adultos submetidos a transplante renal. Um modelo de Markov foi desenvolvido para estimar os custos e os desfechos de cada tratamento a partir do 4º até o 10º ano após o transplante renal^15^.

O modelo pode rastrear os eventos mais relevantes dos pontos de vista clínico e econômico após o transplante renal, incluindo rejeição aguda, infecção por CMV, perda do enxerto, retorno à diálise, retransplante e óbito. Ele pressupõe que os pacientes iniciem a imunossupressão imediatamente após o transplante. Os pacientes entram no modelo em bom estado de saúde com um rim funcionante. Ao longo dos ciclos anuais de simulação, os pacientes podem evoluir para diferentes estados de saúde (infecção por CMV, rejeição aguda, perda do enxerto e óbito). O óbito é considerado um desfecho possível em todos os estados de saúde do modelo. Se o paciente não for a óbito, ele se recuperará. Pacientes que perdem seus enxertos devem retornar à diálise até receber um novo transplante ou falecer (Figura 1).

**Figura 1:** Estrutura do modelo de eventos relacionados à saúde em receptores de transplante renal.

A ocorrência anual de desfechos clínicos dentro de cada grupo de tratamento foi avaliada com base em dados do mundo real até 4 anos após o transplante (Tabela 3).

**Tabela 3:** Incidência de eventos clínicos incluídos no modelo de Markov

N (%)	Ano 1		Ano 2		Ano 3		Ano 4+	
	Sem indução	r-ATG	Sem indução	r-ATG	Sem indução	r-ATG	Sem indução	r-ATG
Infecção/doença por CMV	127 (27,3)	157 (33,7)	–	–	–	–	–	–
Rejeição aguda	236 (50,6)	64 (13,7)	–	–	–	–	–	–
Perda do enxerto (com RA anterior)	15 (3,2)	6 (1,3)	4 (0,9)	1 (0,2)	4 (0,9)	2 (0,4)	3 (0,6)	3 (0,6)
Perda do enxerto (sem RA anterior)	6 (1,3)	7 (1,5)	1 (0,2)	2 (0,4)	5 (1,1)	6 (1,3)	2 (0,4)	6 (1,3)
Óbito com enxerto funcionante	7 (1,5)	13 (2,8)	4 (0,9)	2 (0,4)	2 (0,4)	5 (1,1)	4 (0,9)	3 (0,6)
Óbito após perda do enxerto	4 (19,1)	0	1 (4,8)	1 (7,7)	1 (4,8)	1 (7,7)	3 (14,3)	2 (15,4)
Retransplante	0	0	1 (4,8)	0	0	0	2 (9,5)	0

Abreviaturas: r-ATG: globulina antitimócitos de coelho; CMV: citomegalovírus; RA: rejeição aguda.

Após 4 anos do transplante renal, consideramos para o modelo de Markov as incidências de acordo com referências bibliográficas, conforme descrito na Tabela S2. As incidências anualizadas foram calculadas por meio de um ajuste pelo modelo de Poisson com a fórmula 1 − (1 − λ)^(1/*t*), em que λ é a incidência na referência e t é o tempo^13,16,17^.

Para cada ano de simulação, os pacientes foram alocados aos estados de saúde do modelo de acordo com as probabilidades apresentadas. Para cada ciclo do modelo, essa distribuição de pacientes por estado de saúde é multiplicada pelo custo do estado e, ao longo dos anos de simulação, esse custo é acumulado para obter o custo total.

Consideramos as mesmas incidências para os dois grupos por não haver referências diferentes na literatura, e a lógica da equivalência estatística pode ser aplicada. Ao final do estudo, o impacto na RCEI entre os grupos é determinado pela incidência e pelo custo cumulativos desde o primeiro ano, quando os desfechos e os custos são fatores determinantes para a diferença em 10 anos.

Foi realizada uma análise univariada de todos os parâmetros influenciados pela variabilidade e incerteza dos dados para estimar a confiabilidade dos resultados da avaliação econômica. A análise univariada avalia o modelo matemático de um parâmetro alterando valores dentro de uma faixa do intervalo de confiança de 95%. O principal objetivo é identificar quais parâmetros são críticos para o modelo. O parâmetro com a maior variação dos valores propostos dentro de um IC de 95% indica a variável que exerce a maior influência sobre os resultados da análise.

### Análise de Sensibilidade

Para a tomada de decisão, quantificamos a incerteza no modelo econômico e identificamos as variáveis que impulsionam essa incerteza; portanto, realizamos uma análise de sensibilidade probabilística. O modelo é recalculado utilizando uma simulação de Monte Carlo de segunda ordem na análise de sensibilidade probabilística. O programa seleciona aleatoriamente um valor para cada parâmetro a partir de uma determinada distribuição, uma distribuição beta para as probabilidades de transição. Todos os custos considerados nas análises foram variados em ±10%, utilizando distribuições gama. Embora arbitrária, essa variação é considerada adequada para abranger a variação média de todos os parâmetros de custo utilizados no modelo. O modelo foi executado dez mil vezes, e os resultados são apresentados em um plano de dispersão de custo-efetividade^18,19^.

## Resultados

### Análise de Custo-Efetividade

A análise de custo-efetividade dos episódios de rejeição aguda evitados no primeiro ano após o transplante revelou que o grupo com indução de r-ATG foi mais efetivo. Esse grupo evitou 172 episódios de rejeição aguda, mas a um custo adicional de US$ 68.793,52 (Tabela 4).

**Tabela 4:** Análise de custo-efetividade dos episódios de rejeição aguda evitados no primeiro ano após o transplante renal

	Sem indução (N = 466)	r-ATG (N = 466)	Incremental
Custos Totais (US$)	1.601.209,09	1.670.002,61	68.793,52
Efetividade (episódios de RA)	236	64	172
RCEI (US$ por episódio de RA evitado)			399,96

Abreviaturas: N: número de pacientes; r-ATG: globulina antitimócitos de coelho; RCEI: razão de custo-efetividade incremental; RA: rejeição aguda.

A análise de custo-efetividade da sobrevida do enxerto no primeiro ano após o transplante renal revelou um ganho mínimo de efetividade de 0,01 ano na sobrevida do enxerto no grupo r-ATG e um custo incremental total de US$ 147,50.

A análise de custo-efetividade da sobrevida do enxerto em longo prazo revelou um ganho de efetividade de 0,06 e 0,16 anos na sobrevida do enxerto no grupo de indução com r-ATG e um custo incremental total de US$ −321,68 e US$ −2.440,62, permanecendo dominante em 4 e 10 anos (Tabela 5).

**Tabela 5:** Análise de custo-efetividade da sobrevida do enxerto em 1, 4 e 10 anos após o transplante renal por paciente

	Sem indução	r-ATG	Incremental
**Ano 1**			
Custos Totais (US$)	3.436,11	3.583,61	147,50
Efetividade (sobrevida do enxerto, anos)	0,93	0,94	0,01
RCEI (US$ por ano de sobrevida do enxerto economizado)			14.750,00
**Ano 4**			
Custos Totais (US$)	12.052,15	11.730,47	−321,68
Efetividade (sobrevida do enxerto, anos)	3,19	3,25	0,06
RCEI (US$ por ano de sobrevida do enxerto economizado)			Dominante
**Ano 10**			
Custos Totais (US$)	27.105,66	24.665,04	−2.440,62
Efetividade (sobrevida do enxerto, anos)	5,98	6,14	0,16
RCEI (US$ por ano de sobrevida do enxerto economizado)			Dominante

Abreviaturas: r-ATG: globulina antitimócitos de coelho; RCEI: razão de custo-efetividade incremental.

A análise de sensibilidade unidirecional no ano 1 mostrou que a probabilidade de rejeição aguda (grupo sem indução), infecção/doença por CMV (grupo sem indução) e custos de r-ATG foram as variáveis mais críticas que influenciam o resultado (Tabela S3). Essa análise também mostrou que a probabilidade de infecção/doença por CMV (grupo r-ATG) e a probabilidade de rejeição aguda (grupo sem indução) foram as variáveis mais importantes no ano 4 e a probabilidade de infecção/doença por CMV (grupo r-ATG) foi a variável mais importante no ano 10 (Tabela S3).

### Análise de Sensibilidade Probabilística

A análise de sensibilidade probabilística para episódios de rejeição aguda evitados no primeiro ano após o transplante renal, representada pelas curvas de aceitabilidade de custo-efetividade ou disposição a pagar, mostrou que a probabilidade de custo-efetividade aumenta significativamente a partir de uma disposição a pagar de cerca de US$ 600 e a partir de aproximadamente US$ 1.111; ou seja, há 100% de probabilidade de que a r-ATG seja custo-efetiva em comparação com o grupo sem indução (Figura 2).

**Figura 2:** Curva de aceitabilidade de custo-efetividade para episódios de rejeição aguda evitados no primeiro ano após o transplante renal.

Da mesma forma, a análise de sensibilidade probabilística para a sobrevida do enxerto 4 anos após o transplante renal mostrou que, a partir de uma disposição a pagar de aproximadamente US$ 6.000, existe uma probabilidade superior a 80% de que a r-ATG seja custo-efetiva em comparação ao grupo sem indução. (Figura 3A). Por fim, a análise correspondente para a sobrevida do enxerto em 10 anos após o transplante renal mostrou que, a partir de uma disposição a pagar de aproximadamente US$ 8.000, há mais de 80% de probabilidade de que a r-ATG seja custo-efetiva em comparação ao grupo sem indução (Figura 3B).

**Figura 3:** **A.** Curva de aceitabilidade de custo-efetividade da sobrevida do enxerto em 4 anos após o transplante renal. **B.** Curva de aceitabilidade de custo-efetividade da sobrevida do enxerto em 10 anos após o transplante renal.

### Análise Anual de Custos

A Figura 4 descreve a análise anual de custos para os desfechos em ambos os grupos. As primeiras barras escuras representam o grupo r-ATG, enquanto as barras claras representam o grupo sem indução. Conforme descrito a seguir, os custos do sistema de saúde local para manter os transplantes renais funcionais e para complicações como rejeição aguda e infecção por CMV foram mais elevados no primeiro ano após o transplante. Nos anos subsequentes, o perfil de custos diferiu devido a eventos de perda do enxerto, como o retorno do paciente à diálise, que apresentou o principal desfecho com impacto no orçamento do sistema de saúde.

**Figura 4:** Custos anuais para desfechos no grupo r-ATG versus grupo sem indução. As barras de cor escura representam o grupo r-ATG e as barras de cor clara representam o grupo sem indução.

## Discussão

Os achados deste estudo têm relevância significativa para os tomadores de decisão, uma vez que a escolha da terapia de indução pode influenciar tanto a eficácia quanto os custos a curto e longo prazo após o transplante renal. Realizou-se uma análise farmacoeconômica do mundo real para discutir uma nova estratégia de terapia de indução com dose única de 3 mg/kg de r-ATG em comparação à ausência de indução em receptores de transplante renal com baixo risco de perda do enxerto.

Evidências do mundo real têm o potencial de impulsionar pesquisas sobre sistemas de saúde e fornecer evidências para o controle efetivo de estudos futuros, esclarecendo de que forma fatores como ambientes clínicos, prestadores de serviços de saúde e atributos do sistema de saúde influenciam os efeitos e desfechos do tratamento. Ao aplicar essas evidências, é possível obter *insights* pertinentes a populações mais amplas, superando o que poderia ser alcançado em ambientes de pesquisa especializados. Até o momento, não existe uma análise farmacoeconômica do mundo real sobre o uso da terapia de indução com timoglobulina em receptores de transplante renal no Brasil.

O objetivo deste estudo foi determinar a razão de custo-efetividade incremental relativa à prevenção de episódios de rejeição aguda dentro do primeiro ano pós-transplante, bem como a razão de custo-efetividade incremental relacionada à sobrevida do enxerto renal em 1, 4 e 10 anos pós-transplante, sob a perspectiva do sistema público de saúde brasileiro.

O grupo de indução com r-ATG demonstrou maior efetividade ao evitar 172 episódios de rejeição aguda. A razão de custo-efetividade incremental para esse desfecho foi de US$ 14.750 por episódio de rejeição aguda evitado. A análise de sensibilidade, representada pela curva de aceitabilidade de custo-efetividade, mostrou que, com uma disposição a pagar de aproximadamente US$ 1.111, houve uma probabilidade de 100% de que a r-ATG fosse custo-efetiva em comparação ao grupo sem indução. No Brasil, não existem pesquisas dedicadas à definição de um ponto de corte para a disposição a pagar. Entretanto, a diretriz local recomenda que a análise inclua intervalos de curva de aceitabilidade até o valor de uma a três vezes o produto interno bruto (PIB) per capita do país por QALY. No Brasil, o último valor do PIB per capita foi de US$ 7.946,87, refletindo uma faixa aceitável de disposição a pagar por episódio de rejeição aguda evitado^20^.

A análise de custo-efetividade da sobrevida do enxerto revelou um ganho de efetividade de 0,01, 0,06 e 0,16 anos na sobrevida do enxerto no grupo de indução com r-ATG, respectivamente em 1, 4 e 10 anos após o transplante, sendo dominante nos períodos de 4 e 10 anos. A análise de sensibilidade mostrou uma disposição a pagar de aproximadamente US$ 6.000 e US$ 8.000 para uma probabilidade de 80% de que o uso de r-ATG seja custo-efetivo.

A análise de sensibilidade unidirecional no ano 1 mostrou que a rejeição aguda e a infecção/doença por CMV são as variáveis mais críticas no modelo, relacionadas aos riscos e benefícios da terapia com r-ATG.

No Brasil, o transplante renal é reembolsado por meio de um valor fixo para um pacote de serviços, incluindo cirurgia, materiais hospitalares e medicamentos; no entanto, o montante não cobre todos os custos envolvidos. Dois tipos de terapia de indução estão listados nesse reembolso: r-ATG (Thymoglobulina^®^) e basiliximabe (Simulect^®^), um anticorpo anti-receptor de IL-2. De acordo com a prática local e as evidências científicas, a r-ATG é utilizada em receptores de transplante renal com baixo e alto risco de perda do enxerto.

Análises anteriores investigaram outros aspectos farmacoeconômicos da indução com timoglobulina. Um estudo realizado nos Estados Unidos avaliou, sob a perspectiva dos pagadores, o custo-efetividade incremental entre vários agentes e abordagens para o tratamento imunossupressor precoce em receptores estratificados por risco: sem indução, IL2-RA, r-ATG e alemtuzumabe. Gharibi et al.^21^ estimaram custos cumulativos, sobrevida do enxerto e a razão de custo-efetividade incremental (RCEI - custo por ano adicional de sobrevida do enxerto) dentro de três anos após o transplante entre 19.450 receptores de transplante renal de doador falecido, com o Medicare como principal pagador de 2000 a 2008^19^. Eles segmentaram a coorte do estudo em indivíduos de alto risco (idade > 60 anos, painel de reatividade de anticorpos > 20%, etnia afro-americana, KDPI > 50%, tempo de isquemia fria > 24 horas) e indivíduos de baixo risco (aqueles sem fatores de risco, constituindo 15% da coorte). Os principais achados revelaram a não indução como a abordagem menos efetiva e mais onerosa. A r-ATG e o alemtuzumabe foram considerados mais custo-efetivos em todos os limiares de disposição a pagar no grupo de baixo risco, especialmente nos limiares mais elevados (US$ 100.000 e US$ 150.000). Notavelmente, o grupo r-ATG apresentou curvas de aceitabilidade de custo-efetividade consideravelmente favoráveis (abrangendo 80% dos receptores) em ambos os grupos de risco no limite de US$ 50.000 (excluindo aqueles com idade > 60 anos). Além disso, somente a indução com r-ATG demonstrou um benefício de sobrevida do enxerto em relação à categoria sem indução (razão de risco de 0,91; intervalo de confiança de 95% de 0,84 a 0,99) em uma análise de regressão multivariada de Cox^21^.

Morton et al.^22^ avaliaram a razão de custo-efetividade da terapia de indução com anticorpo anti-receptor de IL-2 (basiliximabe) em comparação com a terapia padrão sem indução ou anticorpo policlonal (r-ATG). O desfecho de efetividade definido nesse estudo foi a sobrevida, obtendo anos de vida salvos e anos de vida ajustados pela qualidade (QALYs). Em comparação com a terapia padrão sem indução, o basiliximabe oferece um ganho de 0,21 anos de vida salvos e 1,42 QALYs, com uma economia de custos ao longo de 20 anos de mais de US$ 79.302 por paciente tratado. Os benefícios incrementais do basiliximabe em relação à indução com r-ATG foram de 0,35 anos de vida salvos e 0,20 QALYs, com um custo incremental de US$ 5.144 por paciente. A razão de custo-efetividade incremental do basiliximabe em comparação com a r-ATG foi de US$ 14.803 por anos de vida salvos e US$ 25.928 por QALYs^22^.

Outra pesquisa realizada em três centros alemães avaliou a relação custo-efetividade das terapias de indução com r-ATG versus basiliximabe em receptores de transplante renal. O custo médio total do tratamento por paciente até o primeiro ano após o transplante foi de € 62.075 e € 59.767 para o grupo r-ATG versus basiliximabe (p < 0,01). A terapia com r-ATG apresentou custos de tratamento semelhantes aos do basiliximabe no segundo ano, com uma previsão de economia cumulativa de custos de tratamento de € 4.259 com r-ATG em comparação com o basiliximabe no décimo ano pós-transplante. A média de anos de vida ajustados pela qualidade (QALYs) por paciente para um ano foi de 0,809 versus 0,802 para r-ATG e basiliximabe, respectivamente (p = 0,38), com QALYs cumulativos de 6,161 e 6,065 por paciente no décimo ano^23^.

Este estudo é limitado por seu desenho de centro único, atributos demográficos específicos da população transplantada e características únicas do sistema público de saúde brasileiro. Consequentemente, a generalização dos achados para populações atendidas por outros sistemas de saúde pode ser um desafio. O modelo foi desenhado a partir da perspectiva do SUS e não da perspectiva da instituição. Portanto, os custos não cobertos pelos pacotes de reembolso do SUS não foram considerados. Alguns aspectos importantes dessa população não foram considerados no modelo farmacoeconômico, como a incidência de nefropatia por poliomavírus BK, doença linfoproliferativa pós-transplante (DLPT) e readmissão hospitalar por citopenia. No entanto, a reprodutibilidade e a análise das experiências clínicas e dos desfechos dos pacientes podem ser viáveis dentro de sistemas de saúde locais e estruturas de reembolso em outros países.

Em resumo, uma dose única de 3 mg/kg de r-ATG é uma terapia de indução custo-efetiva, prevenindo episódios de rejeição aguda e conferindo ganho de sobrevida em longo prazo após o transplante.

## Material Suplementar

O seguinte material online está disponível para este artigo:

Tabela s1 – Custos anuais (US$) de imunossupressão por paciente.

Tabela s2 – Incidência de eventos clínicos incluídos no modelo de Markov após 4 anos.

Tabela s3 – Análise univariada.
